# Moving mitochondria – Breathing new signaling into asthmatic airways

**DOI:** 10.1016/j.redox.2018.07.013

**Published:** 2018-07-21

**Authors:** Quyen Nguyen, Sruti Shiva

**Affiliations:** a Division of Pulmonary Allergy and Critical Care Medicine, University of Pittsburgh School of Medicine, Pittsburgh, PA 15261, USA; b Vascular Medicine Institute, University of Pittsburgh School of Medicine, Pittsburgh, PA 15261, USA; c Department of Pharmacology & Chemical Biology, University of Pittsburgh School of Medicine, Pittsburgh, PA 15261, USA; d Center for Metabolism and Mitochondrial Medicine, University of Pittsburgh School of Medicine, Pittsburgh, PA 15261, USA

Within the cells which execute host immunity and inflammation, mitochondria play a critical regulatory role to support cell activation, pro-inflammatory gene expression, and propagation of inflammatory signaling [1], [2]. Beyond controlling cellular energetics, mitochondria further regulate the function of inflammatory cells through redox signaling, triggering the assembly of inflammasome components, and initiating apoptosis. Mitochondrial reactive oxygen species (ROS) are essential for microbial clearance [3], inflammasome activation [4], T-cell activation and cytokine production [5], and regulation of immune tolerance [6]. Improved understanding of mitochondrial regulation of the inflammatory cascade can potentially reveal novel therapeutic targets for pathologic inflammation in human disease.

In airway diseases such as asthma, the bronchial wall is the site of an aberrant inflammatory response, orchestrated by the cells of the immune system, and propagated within the bronchial epithelium and airway smooth muscle. Mitochondria feature prominently both in regulating the inflammation cascade and mediating tissue damage in the airway. This is evidenced by dysmorphic mitochondrial structure in airway [7], [8], reports of altered mitochondrial function in airway epithelial cells [9], and changes in mitochondrial number in asthmatics [10]. While it is becoming increasingly clear that mitochondria can modulate cellular function from within individual cells, the notion that mitochondria can orchestrate intercellular signaling is also gaining traction. For example, mitochondrial components such as DNA and cardiolipin released from cells have been identified as danger signals that trigger NLRP inflammasome assembly [11], [12], [13], and accumulating studies show transfer of mitochondria between different cell types [14].

**Fig. 1:**
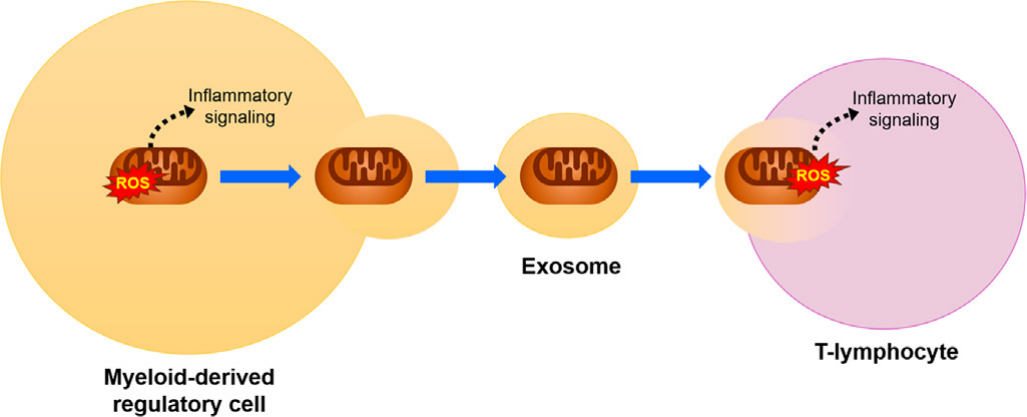
**Exosomal transfer of mitochondria.** Mitochondria from myeloid-derived regulatory cells isolated from bronchoalveolar lavage in human asthma subjects are transferred to T-lymphocytes via exosomes. These mitochondria retain the ability to generate reactive oxygen species (ROS) and are hypothesized to participate in inflammatory signaling.

In this issue of *Redox Biology*, Hough et al. used a range of imaging, flow cytometric, and mitochondria labelling techniques to provide direct evidence for cell-cell trafficking of intact mitochondria in the human airway via exosomes, a subset of extracellular vesicles (EVs) (Fig. 1). The authors first validated the presence of mitochondrial components in EVs isolated from bronchoalveolar lavage (BAL) fluid obtained from human subjects. Asthmatic subjects showed significantly higher numbers of EVs with mitochondrial staining and higher EV mitochondrial DNA content, potentially supporting the notion that enhanced airway inflammation in asthma requires increased mitochondrial recruitment. The authors then derived exosomes from myeloid-derived regulatory cells (MDRCs) purified from BAL fluid. Mitochondria from MDRCs tracked to MDRC-derived exosomes, and importantly, maintained a membrane potential – indicative of preserved bioenergetics function. Considering MDRCs’ role in modulating T-cell function within the inflammatory cascade, the authors then performed a series of experiments to show that T-cells were capable of receiving mitochondria from MDRCs via exosomes to form a mitochondrial network. Autologous peripheral CD4^+^ T-cells were co-cultured with BAL exosomes or MDRC-derived exosomes, and in both conditions, labelled exosomal mitochondria tracked to T-cells and merged with the mitochondrial network already present in the T-cells. Furthermore, MDRC-exosomal mitochondria which transferred to T-cells were capable of promoting mitochondrial ROS, suggesting that they were intact and functional. Taken together, these experiments illustrate a novel mechanism by which mitochondrial signaling can traverse beyond a single cell to potentially coordinate the actions of multiple cell types and regulate cell to cell inflammatory signaling.

Indeed, work in other models has revealed a number of ways in which mitochondria are exchanged between cells to accomplish diverse purposes [14], such as repletion of mitochondrial genetic material, cell rescue [15], [16], immune activation [17], [18], [19], and transmitophagy [20]. To date, mitochondria have been shown to transfer intercellularly via tunneling nanotubes, vesicles, extracellular ejection, and cytoplasmic fusion [14]. For example, mitochondria from stem cells were shown to transfer to injured alveolar epithelial cells through nanotubes, thereby restoring ATP levels and surfactant secretion by the injured cells [21]. Mitochondria can therefore potentially mediate numerous processes within and between cells, however the precise role of mitochondrial transfer in specific human disease states remains largely undefined. Therefore, the investigation undertaken by Hough et al. lays important groundwork to directly establish exchange of intact mitochondria between two cell types relevant to asthma pathogenesis, which were obtained from the human airway.

The importance of studying mitochondrial physiology in human tissue cannot be overstated. Thus, the demonstration of intercellular mitochondrial transfer between immune cells in the human airway to circulating lymphocytes represents a powerful and highly translatable research tool. Recognizing the utility of novel methodologies for study of viable human mitochondria, our group has proposed and validated measurement of platelet bioenergetics as a biomarker in several human disease states, including asthma [22], [23], [24]. While the widespread mitochondrial abnormalities in asthma suggests systemic bioenergetic dysfunction that can be detected in circulating platelets, there exists evidence that platelets also play a direct role in airway inflammation [25], [26]. Platelets, which contain functional mitochondria, can be found in their activated state in BAL from asthmatic subjects [27], and interestingly, are capable of shedding vesicles and extruding mitochondria which modulate the inflammatory response [28]. Taken together with the work of Hough et al., an interesting future direction exists to explore whether intercellular transfer of abnormal platelet mitochondria to other cells within the airway contributes to asthma pathophysiology.

In summary, mitochondria play a central role in cell signaling in inflammatory diseases such as asthma. The investigation performed by Hough et al. provides direct evidence for cell-cell trafficking of intact mitochondria in the human airway, which is pertinent to the pathogenesis of asthma and potentially other airway inflammatory diseases, but also has wider implications for the study of human mitochondrial physiology.
